# Incidence and trends of low back pain hospitalisation during military service – An analysis of 387,070 Finnish young males

**DOI:** 10.1186/1471-2474-10-10

**Published:** 2009-01-19

**Authors:** Ville M Mattila, Petri Sillanpää, Tuomo Visuri, Harri Pihlajamäki

**Affiliations:** 1 Centre for Military Medicine, Lahti and Helsinki, P.O.Box 50, 00301 Helsinki, Finland; 2 Hämeenlinna Central Hospital, Hämeenlinna, Finland; 3 Department of Medical Services, Defence Staff, Finnish Defence Forces, P.O.Box 50, 00301 Helsinki, Finland

## Abstract

**Background:**

There is evidence that low back pain (LBP) during young adulthood and military service predicts LBP later in life. The purpose of this study was to investigate the incidence and trends of LBP hospitalisation among Finnish military conscripts.

**Methods:**

All male conscripts performing their compulsory military service during 1990–2002 were included in the study population. Altogether 387,070 military conscripts were followed throughout their six-to-twelve-month service period. Data on LBP hospitalisations were obtained from the National Hospital Discharge Register.

**Results:**

Altogether 7,240 LBP hospitalisations were identified among 5,061 (1.3%) male conscripts during the study period. The event-based incidence of LBP hospitalisation was 27.0 (95% confidence interval (CI): 25.7–28.2). In most cases, the diagnosis was unspecified LBP (*n *= 5,141, 71%) followed by lumbar disc disorders (*n *= 2,069, 29%). Hospitalisation incidence due to unspecified LBP was 19.1 per 1,000 person-years (95% CI: 18.3 to 20.4), and 7.8 per 1,000 person-years (95% CI: 6.7 to 8.3) due to lumbar disc disorders. The incidence of unspecified LBP remained unaltered, while hospitalisation due to lumbar disc disorders declined from 1993 onwards.

**Conclusion:**

Although conscripts accepted into military training pass physician-performed examinations as healthy, young adults, LBP hospitalisation causes significant morbidity during military service.

## Background

High prevalence rates of low back pain (LBP) have been reported among working-age populations [1,2]. The prevalence of LBP is highest in middle age, but an increasing occurrence has been observed among adolescents and young adult populations [3-6]. It has been estimated that by the age of 20 approximately fifty percent of people have suffered from LBP at least once [7], while the prevalence of sciatic pain has varied between 1.8% [8] and 4% [9]. There is evidence that LBP during young adulthood and military service predicts LBP later in life [10], and may lead to chronic disability [1,2]. Although LBP has been studied widely in active duty soldiers, and estimated to cause a loss of billions of dollars annually in the United States [11], morbidity and incidence of LBP hospitalisations have not been described in military conscript populations.

The increasing prevalence of overweight and obesity and the overall trend of declining physical activity among adolescents and young adults have raised substantial concerns in developed countries [12,13]. A previous study by Santtila et al. [12] from Finland showed a significant deterioration in physical fitness and, simultaneously, an increase in body weight among Finnish conscripts during the past three decades. An interesting question is how these changes affect the prevalence of LBP. Based on existing knowledge, the association between overweight and LBP among adolescents and young adults is weak [14-17], but overweight is associated with clinically defined sciatica [18]. Similarly, the association between physical activity and LBP has emerged as a controversial issue, since both high [17,19-21] and low levels [22,23] of physical activity have been identified as risk factors for LBP. In addition, a recent review article found no evidence of association between LBP and physical capacity [24].

Military service in Finland is compulsory for all male citizens above 18 years of age, and over 80% of men complete their service period, duration varying from 6 to 12 months. Military service begins with a 9-week basic training period, the content of which is basically the same for all conscripts. The special characteristics of military training include both the intensity and, since one of the main goals of the training is to improve conscripts' physical performance levels, the volume of physical activity which increases linearly during conscription. Due to the obligatory entry to military service in Finland, the epidemiological figures can be quite well generalisable to the young adult male population when mixed study settings involving sports-related or active duty soldiers are avoided.

Given the fact that during military service all conscripts have to use the services of military hospitals, the aims of the present study were to investigate the incidence, nature and secular trends of LBP hospitalisation among Finnish male conscripts during physically strenuous military service in 1990–2002 using the database of the National Hospital Discharge Register (NHDR).

## Methods

### study population

During the first five study years, 1990–1994, military service duration was 9 or 11 months and the starting interval was five months. Since 1995, the length of the compulsory military service period has been 6, 9, or 12 months, and a new batch of conscripts enters twice a year, in January and July (Table 1.). Service duration depends on the assigned position: those being trained to be officers, non-commissioned officers, or specialists for highly demanding duties serve for 12 months, those trained for duties requiring expert skills serve for nine months, and those trained for other rank and file serve for six months. Since 1995, when voluntary military service was made available to female citizens, approximately 1–2% of the conscripts have been women. However, due to the small proportion of females in the military service, women were excluded from the analysis. In general, military service is performed at the age of 19–20, but earlier (18 years) or later (up to 29 years) entry is possible in special cases due to voluntary recruitment or deferment. Annually, approximately 23,000–32,000 conscripts start their service. The total number of conscripts who entered into military service during the study period 1990–2002 was 387,070 conscripts (women excluded). Given that the length of the military service varies between six and 12 months, the total exposure-time during the study period between 1990 and 2002 was 267,700 person-years. The average age of the conscripts was 20 (range 18 to 29) years, average length 178 cm, and average weight 72 kg, yielding a body mass index (BMI) of 23.2 kg/m^2^.

**Table 1:** Changes in coding system and duration of military service during the study period in 1990–2002.

**Change**	**year**
ICD-coding system	1996
Duration of service from 9 and 11 months to 6, 9 or 12 months	1995

Military training begins with a two-month basic training period consisting of 135 hours of varying physical training (17 hours per week), including marching, cycling, drill training, combat training, or other training involving heavy physical loading. During combat training and marching, every conscript usually carries approximately 26 kg (in summer) or 36 kg (in winter) of personal military equipment and, occasionally, an additional 5 kg to 20 kg of team military equipment. In addition, conscripts perform 56 hours (7 hours per week) of other physical exercises such as jogging, team sports or circuit training. After the basic training period, the amount of moderate and high-intensity physical training is slightly reduced (to 15 hours per week).

### low back pain hospitalisation data

LBP hospitalisation data were obtained from the statutory, computer-based National Hospital Discharge Register of Finland (NHDR), which included information on all military conscripts admitted alive to military hospitals as inpatients for the treatment of LBP from January 1^st^, 1990 to December 31^st^, 2002. The main outcome variables were defined as patients hospitalised with the main diagnosis of 1) lumbar and other intervertebral disc disorders with radiculopathy (lumbar disc disorder), and 2) unspecified LBP. The diagnosis in the National Hospital Discharge Register had been coded using the 9th (1990–1995) and 10th (1996–2001) revisions of the International Classification of Diseases (ICD) [25]. LBP hospitalisation was defined in the ICD-10 by codes M51.1 (lumbar and other intervertebral disc disorders with radiculopathy), M54.5 (low back pain), and M54.9 (dorsalgia), and in the ICD-9 by codes 7227C, 3539X, 7242A, 7245A, and 7249X, respectively (Table 1.). For analysis, the diagnoses were further categorised into lumbar disc disorders and unspecified LBP. All persons who started their military service during the study period, regardless of whether they served full term or not, were included in the study. During military service, all conscripts are obligated to use the medical services provided by military hospitals. In case of an emergency visit to a civilian hospital, conscripts are promptly transferred to a military hospital. Approval to use the hospitalisation data was obtained from the Institutional Review Board of the Ministry of Social Affairs and Health.

### statistical analysis

This study was based on register data utilising Finland's system of national identification (ID) numbers. All Finnish citizens are assigned a personal ID-number, which, after approval by the appropriate authority and ethical committee, may be used in scientific research. SPSS 14.0.1 for Windows software was used for the statistical analysis. We calculated the event-based occurrence of LBP hospitalisation by dividing the number of LBP hospitalisations by exposure-time. Incidence and incidence rate-ratio were calculated with 95% confidence intervals (CI). The length of the skewed hospitalisation period was compared between groups using the Mann-Whitney *U*-test.

## Results

During the study period, altogether 7,240 LBP hospitalisations were identified among 5,061 conscripts, the total exposure-time being 267,700 person-years. The proportion of conscripts hospitalised during military service due to LBP was 1.3%. The event-based incidence of LBP hospitalisation was 27.0 (95% CI: 25.7–28.2) per 1,000 person-years. In most cases, the diagnosis was unspecified LBP (*n *= 5,141, 71%) followed by lumbar disc disorders (*n *= 2,069, 29%). The incidence of hospitalisation due to unspecified LBP was 19.1 per 1,000 person-years (95% CI: 18.3 to 20.4), and the incidence of hospitalisation due to lumbar disc disorder was 7.8 per 1,000 person-years (95% CI: 6.7 to 8.3). The overall median length of hospital stay was three (range 1 to 114) days. In conscripts hospitalised due to lumbar disc disorder, the median length of hospital stay was four days compared to three days in those with unspecified LBP (*P *< 0.001). Multiple LBP hospitalisations occurred in 1,113 (22%) conscripts, the maximum number of hospitalisations being 10 during the military service.

### incidence trends

**Figure 1:**
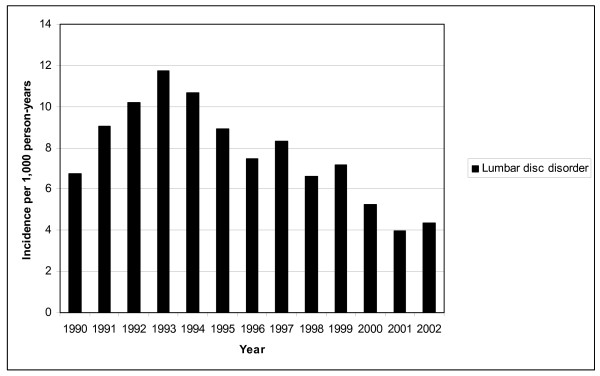
**Incidence of hospitalisation for lumbar disc disorder in Finnish conscripts in 1990–2002**.

**Figure 2:**
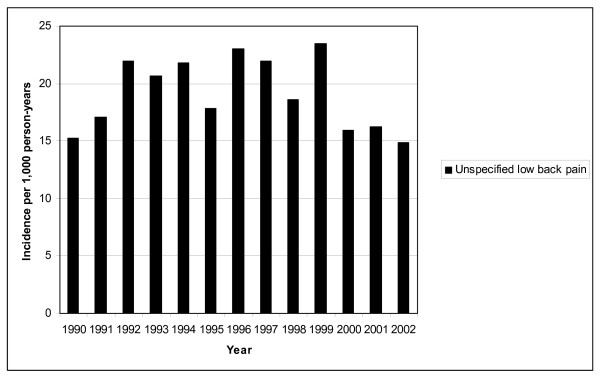
**Incidence of hospitalisation for unspecified low back pain in Finnish conscripts in 1990–2002**.

Figure 1 and 2 show the incidence of lumbar disc disease and unspecified LBP hospitalisations in Finnish conscripts in 1990–2002. The annual number of LBP hospitalisations varied between 366 (in 2002) and 728 (in 1993). Lumbar disc disorders constituted 30% of all LBP-related hospitalisations during the whole 13-year study period. The highest incidence of LBP hospitalisation, 32.2 per 1,000 person-years (95% CI: 29.6–35.3), was seen from 1991 to 1993, and the lowest incidence, 18.9 per 1,000 person-years (95% CI: 17.2–22.1), was seen in 2002. The incidence of hospitalisations due to unspecified LBP did not vary significantly during the study period, being 18.6 per 1,000 person-years (95% CI: 17.3–20.4). A decline in the incidence of hospitalisations due to lumbar disc disorders was found, from 12.3/1,000 person-years (highest) in 1993 (95% CI: 9.6–13.3) to 4.2/1,000 person-years (lowest) in 2001 (95% CI: 3.0–5.0), yielding a relative risk ratio of 0.4 (95% CI: 0.3–0.5).

## Discussion

The principal finding of the present study was that 1.3% of our male conscript study population were hospitalised for LBP during their mandatory military service. The incidence of LBP hospitalisation was 27 per 1,000 person-years and a decline in the incidence of hospitalisation due to lumbar disc disorders was found from 1993 onwards. In most cases, hospitalisation was due to unspecified LBP, while lumbar disc disorders accounted for one third of the hospitalisations. LBP causes significant morbidity during conscription. Military service training involving high-intensity physical activity and higher spinal load offers unique opportunities to identify persons with a proneness to LBP.

Our study has notable strengths. The population at risk consisted of a significant number of individuals obliged to use the medical services provided by the Finnish Defence Forces for the management of LBP. Further, all conscripts had passed two, physician-performed medical examinations when entering military service, on which occasion all patients with severe back diseases, such as significant scoliosis or other congenital back anomalies, severe post-traumatic disorders, rheumatoid arthritis, or ankylosing spondylitis were exempted from duty. With good reason, conscripts meeting all medical requirements can be considered healthy young men. In addition, the accuracy and extensiveness of the NHDR database [26-28] have been shown to be excellent. The accuracy of the incidence figures for conscripts' LBP hospitalisations is superior to that of any civil hospital registers, as the cohort included in this study had no alternative choices such as private clinics to attend due to LPB. Moreover, the authors conclude that the results are very well generalisable to a young healthy male population owing to the compulsory nature of the Finnish military service.

This study has also some weaknesses. Unfortunately, we had no means to identify less severe LBP episodes because of a lack of computer-based outpatient registers for the early 1990s. In addition, due to our relatively long follow-up period, some changes seen in the incidence of LBP hospitalisation may have been caused by changes in hospitalisation policies and not by true variation in the incidence. Although the emphasis in LBP management has shifted towards more active treatment regimen in Finland, this explanation is not presumable, because no changes in the incidence of unspecified LBP were seen.

The proportion of conscripts with LBP hospitalisation (1.3%) may be considered significant for several reasons: the healthy young men comprising our study sample represented 80% of the age group, had passed two medical examinations and successfully met the health requirements set for military service. The incidence of LBP hospitalisation seen in the present study is higher than in previous publications performed in population-based study settings [9]. The hospitalisation rates among conscripts may not be directly comparable to those among recruits or civilians. All conscripts have free health care and obligation to use military hospitals, and thus the threshold of hospitalisation may be somewhat lower in military service than elsewhere. In other words, if a conscript with LBP is not able to stay in garrison despite normal non-steroidal anti-inflammatory drug treatment, he is hospitalised. Another possible explanation to increased hospitalisation rates compared to civilian populations may be the increased levels of physical exercise and lifting. The increased spinal load and the mechanical load on the intervertebral discs may cause lumbar disc herniation. Other mechanisms may include muscle tightness and loss of flexibility resulting in decreased lumbar flexion, which again has shown to be associated with LBP in athletes [29] and in military conscripts [30] too. Another explanation may be the increase in injury rates during military service [31], which may predispose to LBP [19]. A point worth noting is that despite the increasing trend of obesity and deterioration in physical fitness during the study period, no simultaneous increase in LBP hospitalisations was seen. One fifth of the conscripts had more than one LBP-related hospitalisation during the study period. Although we had no opportunity to follow these persons after completion of military service, we may suggest that they have an increased risk of long-term morbidity caused by LBP. Prevention measures should hence be targeted at conscripts with several LBP hospitalisations during their military service.

A notable finding in the present study was the decline in the incidence of LBP hospitalisation during the study period, which was attributed to a reduction in hospitalisations due to lumbar disc disorders. As the decline started in 1993, before the ICD-9 was superseded by the ICD-10 coding system in 1995, this change is not a viable explanation. Changes in military training or equipment do not explain this decline either. Because population-based information on the prevalence of lumbar disc disorders is not available for this period, the authors cannot conclude if the phenomenon results from a real change in the incidence or perhaps from changes in hospitalisation policies. A reduction in the frequency of hospitalisations due to lumbar disc disorders is supported by the findings from a recent Finnish report, in which the overall decline in lumbar disc surgeries amounted to 27% from 1995 to 2005 with simultaneous reduction in the number of hospitalisation days by 13% [32]. The tendency towards more active mobilisation after lumbar disc disorders may in turn partly explain the decline in the overall hospitalisation incidence. However, a significantly longer hospital stay (four vs. three days) was observed due to lumbar disc disorders than due to unspecified LBP.

## Conclusion

Our study sample comprised 391,241 military conscripts representing 80% of a given age group of healthy Finnish males. The incidence of LBP hospitalisation due to lumbar disc disorders underwent a decrease from 1993 onwards. In most cases, hospitalisation was due to unspecified LBP, while lumbar disc disorders accounted for one third of the hospitalisations. Although military conscripts are healthy, physically active young adults, LBP causes significant morbidity in this population. Military service environment offers unique opportunities to prevent morbidity caused by LBP.

## Competing interests

The authors declare that they have no competing interests.

## Authors' contributions

VM drafted the manuscript and performed statistical analysis. PS, TV and HP designed the study. HP conceived of the study, and participated in its design and coordination and helped to draft the manuscript. All authors read and approved the final manuscript.

## Pre-publication history

The pre-publication history for this paper can be accessed here:


